# Editorial: Environmental adaptation for accessibility: a global perspective in the field of disability, rehabilitation and inclusion

**DOI:** 10.3389/fresc.2024.1514744

**Published:** 2024-11-08

**Authors:** Hassan Izzeddin Sarsak, Ernesto Morales, Manigandan Chockalingam

**Affiliations:** ^1^ Occupational Therapy Program, Batterjee Medical College, Jeddah, Saudi Arabia; ^2^ Faculty of Medicine, School of Rehabilitation Sciences, Université Laval, Quebec, QC, Canada; ^3^ Occupational Therapy Discipline, School of Health Sciences, University of Galway, Galway, Ireland

**Keywords:** accessibiity, environmental adaptation, inclusion, persons with disabilities, universal design

**Editorial on the Research Topic**
Environmental adaptation for accessibility: a global perspective in the field of disability, rehabilitation and inclusion

Accessibility within the built environment is a critical and primary concern in urban planning and design (1, 2). Public spaces can only be deemed successful if they provide accessibility for all individuals (3). Accessibility must extend to everyone, regardless of physical abilities or financial resources, as it embodies the freedom and ease for individuals to engage and participate in a variety of activities (4–6). Assessing building accessibility and implementing environmental adaptations are essential initial steps in the process of eliminating barriers to accessibility. Such assessments facilitate the identification of accessibility challenges and potential solutions within existing facilities, thereby fulfilling obligations to universal accessibility standards and ultimately making these spaces accessible for persons with disabilities (PWDs) (7–10). However, there remains a notable lack of studies examining the degree and extent of accessibility in public spaces and the adequacy of accommodations provided.

In recognition of World Health Day and the anniversary of the World Health Organization (WHO), Frontiers in Rehabilitation Sciences has launched a series of research topics aimed at highlighting prominent and emerging practices, concerns and developments in rehabilitation all over the world. Given the scarcity of studies focused on environmental adaptations and accessibility, coupled with the journal's commitment to advancing research on emerging rehabilitation practices, we present a special collection dedicated to environmental adaptations and the utilization of assistive technologies to enhance accessibility for a diverse range of end users worldwide. Our objective was to consider the experiences of various end users and the distinct environments in which these practices and technologies are implemented. Additionally, we seek to underscore the latest advancements in environmental adaptations for accessibility across multiple sectors, including rehabilitation, clinical settings, and social and economic environments.

The eleven articles in this collection speak to six major themes relating to:

1. Advancements in assistive technologies: rehabilitative, clinical, and social environments.
2. End user satisfaction with assistive technologies during rehabilitation.
3. Innovative approaches in the field of environmental adaptations for accessibility in complex public spaces.
4. Advancements in environmental adaptions for accessibility within organizations.
5. Implementation of best practices in the field of assistive technologies during rehabilitation.
6. Emerging trends: environmental adaptations in rehabilitative, clinical, and social environments from a global health perspective.

Through a diverse array of contributions, ranging from original research to systematic reviews, the authors of this special research topic present key findings and advancements in assistive technology from a global perspective, specifically addressing the domain of environmental adaptation for the accessibility of PWDs.

In this special issue, the Ingabire et al. study entitled “*Factors affecting social integration after road traffic orthopaedic injuries in Rwanda*” aimed to identify factors contributing to social integration following road traffic-related orthopedic injuries (RTOI) in Rwanda. This study concluded that the majority of RTOI victims in Rwanda achieved successful reintegration into society; nevertheless, their mobility and community engagement were more significantly impacted compared to other aspects. This study emphasized the importance of early management, effective rehabilitation, and prompt patient discharge from the hospital in facilitating a successful return to everyday life after road traffic-related orthopedic injuries.

The Thériault et al. review article aimed to address the following questions: (1) Which are the current fire evacuation learning strategies used with PWD or seniors? (2) What are the barriers and facilitators for PWD and seniors’ during fire evacuation and learning strategies? (3) What is the existing equipment that could be used with PWD seniors? This study found that the current fire evacuation learning strategies currently used can be grouped into three categories: drills; training; promotion of a fire safety plan. Six types of evacuation equipment were found; however, their use has been scarcely documented. They concluded that safety for seniors during fire evacuation is still an important issue to be improved and increasing awareness and creating new practices and tools that consider the strengths and difficulties of seniors seems to be a promising avenue for improving evacuation.

A scoping review on accessibility and inclusion of people with disabilities in international airports by Gotti et al. is considered as the first step of a broader project supported by Canadian accessibility standards, focusing on enhancing inclusive accessibility in Canadian airports. They concluded that services need to be extensively planned, placing a significant burden on passengers. The disability-centric perspective disregards passengers’ unique needs and capabilities, leading to a sense of dehumanization. The complexity of airport organizations, shared responsibilities, limited communication, training challenges can deter accessibility initiatives and create discomfort during travel. In this study, they reported that they will be accompanying PWD of various profiles through the various stages of their journey, to gather their perceptions of the travel experience. The results will be then used in conjunction with this study to formulate recommendations and solutions for inclusive accessibility.

Ripat et al. study outlines the creation and standardization of an outdoor environment designed to simulate the real-life conditions and obstacles experienced by manual wheelchair (MWC) users in winter. This project aimed to offer several additional potential benefits, supported by the various stakeholders across the study phases that extend beyond creation of a controlled and safe environment for wheelchair users to develop their winter mobility skills. They have concluded that practicing wheelchair skills in this area may assist wheelchair users in gaining confidence which may ultimately translate to increased participation in the community.

The Corcuff et al. study entitled “*Co-design knowledge mobilization tools for universal accessibility in municipalities*” was conducted to develop knowledge mobilization tools tailored to a specific municipal context in Quebec, Canada, to facilitate the implementation of universal accessibility measures by municipal employees. The co-design process employed in this study was organized into four distinct stages, following the Morales model: (1) Exploration (2) Co-Design (3) Validation (4) Development. The stages one and two highlighted the employees’ lack of awareness about universal accessibility issues and their need to have more information and resources about how universal accessibility is encountered in their work. A steering committee co-designed three video vignettes about universal accessibility, the city's action plan and measures included in it. The co-design approach used in this study allowed to observe the non-linear nature of partnership research with an organization as complex as a municipality and showed significant advantages of collaboration between the municipal sector and research.

Derakhshan et al. scoping review was conducted to identify barriers and facilitators to participation in adaptive outdoor physical activity (PA) and identify suggestions for adaptive outdoor PA design. This study identified gaps in knowledge about facilitators and barriers to outdoor adaptive PA and in the design of interventions addressing them. They concluded that future research should focus on the strategies addressing these gaps by involving individuals with mobility disability in designing interventions to gain a better insight into their needs.

Mwaka et al. review study aimed to examine barriers and facilitators of public transport use among people with different types of disabilities during the entire travel chain and to explore perceived self-efficacy and satisfaction related to public transport experiences among PWD. This study showed that people with various forms of disability continue to encounter difficulties in accessing and using public transit throughout the entire travel chain, due to many physical and social barriers despite the adoption and implementation of the Convention on the Rights of Persons with Disabilities (CRDP). This review identified the physical and social barriers and facilitators that can occur in different links of the travel chain and highlighted issues related to lack of confidence and decreased satisfaction when PWD and older adults are using public transport. The identification of barriers and facilitators to the use of public transport by PWD is an important step that may help policy makers and transport operators around the world to develop and implement interventions to facilitate access, use and inclusion of this mode of transport, as the experiences of PWD when using this mode of transport have an impact on their well-being. The results of this scoping review could lead to a better understanding of the potential barriers and facilitators to the use of public transport by people with various disabilities and how negative or positive experiences throughout the travel may influence their self-efficacy and satisfaction.

In this issue, Kuo et al. article analyzed the risks and benefits that video games may present to individuals with disabilities. Their findings showed that individuals with disabilities are most at risk from excessive video game use, leading to increased aggression, sedentary behavior, and negative impact on academic performance. Identified benefits included promoting physical rehabilitation and psychological well-being, improving cognitive abilities and emotional regulation, and utility in promoting exercises, and managing chronic pain. This article presented a number of strategies and resources to help guide individuals with disabilities, educators, practitioners, and researchers in maximizing the benefits of video games while controlling the risks.

Ramôa et al. study aimed to enhance the accessibility of 2D information for individuals with individuals with blindness or visual impairment (BVI). The rapid advancements in 2D tactile readers and 2D pin-matrix displays hold immense potential for revolutionizing information accessibility for individuals with visual impairments. Based on this study, the Sonoice navigation user interface has emerged as a notable solution, achieving higher levels of efficiency compared to the sonar and voice methods. Their findings highlighted the potential of navigation strategies to enhance the accessibility and usability of tactile graphics for individuals with visual impairments, emphasizing the importance of incorporating such user interfaces in future design and development efforts. They concluded that understanding individual preferences and tailoring the user interface accordingly is essential for optimizing user satisfaction and effectiveness in tactile graphics exploration. Furthermore, as tactile graphics readers and 2D refreshable braille hardware technology continue to grow, it is essential to define optimal user interface standards and expand the capabilities and application domains, further empowering individuals with visual impairments.

Ruiz-Rodrigo et al. study entitled “*experiencing accessibility of historical heritage places with individuals living with visible and invisible disabilities*” was conducted to explore the experiences of people with visible and invisible disabilities when visiting heritage sites considering accessibility issues. The barriers identified by participants in this study were diverse and differ according to the person and the type of disability. However, social and leisure activities were particularly limited, despite the strategies developed by some participants. Participants in the study demonstrated an interest in accessing to heritage places, therefore it seems essential to consider the needs of people with disabilities when developing accessibility solutions, and to seek a balance between preserving heritage and promoting inclusive and equitable access for all.

The “*Enhancing Shared Street Accessibility in Heritage Sites for Individuals with Visual Disabilities: A Canadian Perspective*” study by Lakoud et al. explored how shared streets can be adapted to be more inclusive while respecting the integrity of historical environments. The objective of this study was to explore and propose practical solutions to enhance the accessibility of shared streets for individuals with visual disabilities within heritage sites, with a particular focus on preservation requirements. The study concluded that shared streets can be made more accessible for individuals with visual disabilities by adopting a modular design approach that integrates tactile cues and adaptable urban furniture. These solutions ensure that accessibility and safety can coexist with heritage preservation, promoting inclusivity in public spaces. The research highlights the importance of stakeholder engagement in the design process and offers a replicable framework for improving accessibility in heritage sites globally. However, further field testing is needed to assess the feasibility and acceptance of these solutions within the regulatory constraints of heritage environments.

## Final word

To enhance accessibility, environmental adaptations, and the implementation of assistive technologies, it is essential to foster collaboration among end users, healthcare professionals, engineers, architects and policymakers. Interdisciplinary teams must work together to create environments that are accessible to all individuals with disabilities, thereby addressing the needs of this vulnerable population on a global scale. Moreover, conducting qualitative research that incorporates the valuable and meaningful perspectives and insights of PWDs is vital. This approach ensures that the voices of those affected are heard, ultimately leading to more effective and inclusive solutions.

This issue also contributes to another of the fundamental tasks of improving accessibility in the world: raising awareness and educating. The dissemination of knowledge is an invaluable tool in educating us about the great importance of creating accessible spaces for all in the diverse and pluralistic world in which we live.
